# BSA-Seq Discovery and Functional Analysis of Candidate Hessian Fly (*Mayetiola destructor*) *Avirulence* Genes

**DOI:** 10.3389/fpls.2020.00956

**Published:** 2020-06-25

**Authors:** Lucio Navarro-Escalante, Chaoyang Zhao, Richard Shukle, Jeffrey Stuart

**Affiliations:** ^1^ Department of Entomology, National Coffee Research Center, Manizales, Colombia; ^2^ Department of Botany and Plant Sciences, University of California, Riverside, Riverside, CA, United States; ^3^ USDA-ARS and Department of Entomology, Purdue University, West Lafayette, IN, United States; ^4^ Department of Entomology, Purdue University, West Lafayette, IN, United States

**Keywords:** plant immunity, effector protein, resistance gene, plant gall, genome sequencing, wheat

## Abstract

The Hessian fly (HF, *Mayetiola destructor*) is a plant-galling parasite of wheat (*Triticum* spp.). Seven percent of its genome is composed of highly diversified signal-peptide-encoding genes that are transcribed in HF larval salivary glands. These observations suggest that they encode effector proteins that are injected into wheat cells to suppress basal wheat immunity and redirect wheat development towards gall formation. Genetic mapping has determined that mutations in four of these genes are associated with HF larval survival (virulence) on plants carrying four different resistance (*R*) genes. Here, this line of investigation was pursued further using bulked-segregant analysis combined with whole genome resequencing (BSA-seq). Virulence to wheat *R* genes *H6*, *Hdic,* and *H5* was examined. Mutations associated with *H6* virulence had been mapped previously. Therefore, we used *H6* to test the capacity of BSA-seq to map virulence using a field-derived HF population. This was the first time a non-structured HF population had been used to map HF virulence. *Hdic* virulence had not been mapped previously. Using a structured laboratory population, BSA-seq associated *Hdic* virulence with mutations in two candidate effector-encoding genes. Using a laboratory population, *H5* virulence was previously positioned in a region spanning the centromere of HF autosome 2. BSA-seq resolved *H5* virulence to a 1.3 Mb fragment on the same chromosome but failed to identify candidate mutations. Map-based candidate effectors were then delivered to *Nicotiana* plant cells *via* the type III secretion system of *Burkholderia glumae* bacteria. These experiments demonstrated that the genes associated with virulence to wheat *R* genes *H6* and *H13* are capable of suppressing plant immunity. Results are consistent with the hypothesis that effector proteins underlie the ability of HFs to survive on wheat.

## Introduction

The Hessian fly (HF, *Mayetiola destructor*) is an economically important, gall-forming, insect pest. It has a gene-for-gene relationship with its host plant, wheat (*Triticum* spp.). Recent investigations involving HF *Resistance* (*R*) genes *H13* and *H9* in wheat illustrate this relationship (Stuart, 2015): *H13* normally prevents HF larvae from galling wheat. These “*H13*-avirulent” larvae die as first instars on *H13*-plants. In contrast, “*H13*-virulent” HF larvae overcome this resistance; they both survive and gall *H13*-plants. This ability to survive and gall (*H13* virulence) is conditioned by recessive null mutations in a single HF gene, called *Avirulence* (*Avr*) gene *vH13* (Aggarwal et al., 2014). These *vH13* mutations are *H13*-specific. They do not, for example, allow plant galling and HF survival (virulence) on wheat plants carrying *R* gene *H9*. Instead, larvae that defeat *H9*-resistance are homozygous for recessive null mutations in a different *Avr* gene, *vH9* (Zhao et al., 2015). Wheat has at least 35 dominant, simply inherited, resistance (*R*) genes that prevent “avirulent” HF larval survival and plant galling (Miranda et al., 2010). The gene-for-gene hypothesis predicts that 35 different *Avr* genes correspond to each one of these *R* genes.

Similar gene-for-gene relationships exist between plants and plant pathogens (Harris et al., 2010). The study of these interactions has revealed two levels of defense in the plant immune system (Jones and Dangl, 2006). Basal plant immunity defends against non-adapted organisms. Highly adapted plant parasites use effector proteins to defeat this basal defense. To counter these parasites, plants have a second level of defense called Effector-Triggered Immunity (ETI). ETI uses *R*-gene-encoded proteins (R proteins) that recognize, either directly or indirectly, the presence of specific effectors. Upon effector detection, plant cells initiate a defense response that limits plant damage and infection. Natural selection then favors pathogens that have either masked or modified the effector beyond R-protein recognition or have lost the effector completely. This suggests that *Avr* genes are simply parasite genes that encode the effectors recognized by plant R proteins (Hogenhout et al., 2009).

Therefore, one hypothesis is that ETI underlies the HF-wheat gene-for-gene interaction. The corollary is that the HF uses effector proteins to defeat basal plant immunity. Additional evidence in favor of these hypotheses exist in both the plant and the insect. With respect to the plant, most *R* genes belong to gene families that encode proteins with nucleotide-binding (NB) and leucine-rich repeat (LRR) domains (Jones and Dangl, 2006). As natural selection has presumably favored their evolution in response to parasite adaptation, these are among the most prevalent and diverse genes in plant genomes. The genome of *Aegilops tauschii*, one diploid progenitor of hexaploid bread wheat (*T. aestivum*), contains over 1200 NB-LRR genes (Jia et al., 2013). Although the sequence of a HF *R* gene in wheat has yet to be published, mapping data indicates that they reside in clusters of NB-LRR genes (Gill et al., 1987; Raupp et al., 1993; Kong et al., 2005; Liu et al., 2005b; Liu et al., 2005c; Sardesai et al., 2005; Kong et al., 2008; Miranda et al., 2010).

With respect to the insect, hundreds of HF genes (seven percent of the HF genome) encode putative effectors. The majority of these are members of large, diverse gene families that were originally discovered as signal peptide-encoding transcripts in first-instar larval salivary glands (Chen et al., 2004; Chen et al., 2008; Chen et al., 2010). Some of these have been identified in HF-infested wheat tissue (Zhao et al., 2015; Wang et al., 2018). Like effector encoding genes in plant parasites (Hogenhout et al., 2009), these HF genes are experiencing diversifying selection (Chen et al., 2008; Zhao et al., 2015), presumably to remain adapted to wheat. Moreover, HF *Avr* gene mapping has shown a correspondence between HF *Avr* genes and putative effector-encoding genes (Stuart, 2015).

Here, we describe experiments that further tested this correspondence. Additional HF *Avr* gene mapping was performed using bulked-segregant analysis (Giovannoni et al., 1991; Michelmore et al., 1991) in combination with whole genome resequencing (BSA-seq). BSA uses pools of genomic DNA collected from individuals segregating for a trait of interest to identify polymorphic DNA markers linked to that trait. BSA-seq sequences pools of genomic DNA to identify linked single nucleotide polymorphisms (SNPs) and then directly positions those SNPs in the genome. BSA-seq has been successfully applied to gene mapping and identification in yeast (*Saccharomyces cerevisiae*) (Pomraning et al., 2011; Swinnen et al., 2012), zebrafish (*Danio rerio*) (Leshchiner et al., 2012), *Arabidopsis thaliana* (Austin et al., 2011; Schneeberger, 2014), rice (*Oryza sativa*) (Abe et al., 2012; Takagi et al., 2013); fruit fly (*Drosophila melanogaster*) (Bastide et al., 2013) and the malaria mosquito (*Anopheles gambiae*) (Redmond et al., 2015). It was also used to locate mutations in the brown planthopper (*Nilaparvata lugens*) *Avr* gene *vBph1* that defeat the *Bph1*-resistance in rice (*Oryza sativa*) (Kobayashi et al., 2014). Here, we were interested in three separate HF traits: virulence (defined as larval survival and plant-galling) to *H6*-, *Hdic*- and *H5*-resistant wheat seedlings. Virulence to *H6*, which had been mapped previously (Zhao et al., 2015), tested the accuracy of BSA-seq in the HF. We then mapped virulence to *Hdic* and *H5*.

To test putative HF effectors for plant immune suppression, we employed an assay that uses *Burkholderia glumae* bacteria, and the effector detector vector (pEDV) system to deliver HF candidate Avr proteins to *Nicotiana tabacum* and *N. benthamiana via* the bacterial type III secretion system (T3SS) (Sohn et al., 2007; Fabro et al., 2011). *B. glumae* is a bacterial rice pathogen that causes a rapid, localized cell death (a hypersensitive reaction, HR) in non-host *N. tabacum* and *N. benthamiana*. The pEDV system uses the type III secretion system (T3SS) to mediate foreign effector protein translocation into plant cells (Sohn et al., 2007; Sohn et al., 2014). Combined, *B. glumae* and pEDV effector protein translocation in *Nicotiana* plant cells enables the discovery of effectors with plant-immune suppression activity. The *B. glumae*/pEDV/*Nicotiana* system has been used to test effectors from the rice blast fungus *Magnaporthe oryzae* (Sharma et al., 2013), the false smut *Ustilaginoidea virens* (Zhang et al., 2014), the pathogenic fungus *Lasiodiplodia theobromae* (Yan et al., 2018) and the root knot nematode *Meloidogyne incognita* (Shi et al., 2018a). Here, we examined proteins encoded by candidate HF *Avr* genes *vH13*, *vH6,* and *vHdic*.

## Materials and Methods

### BSA-Seq Analysis

BSA-seq was used to perform genomic mapping of virulence to three HF *R* genes: *H6, Hdic,* and *H5*. To do this, DNA bulks were prepared using DNA isolated from individuals segregating for virulence. This required that populations segregating for virulence and avirulence to each *R* gene had to be identified and that individuals within these populations had to be separately genotyped. Virulence to each wheat *R* gene examined presented different challenges. The solution was to prepare separate HF populations for each *R* gene. A description of each population is presented below, followed by a description of whole-genome sequencing and data analysis. Later, we describe the preparation of PCR-based markers that were used to improve genetic resolution.

#### Wheat *R* Genes and Insect Mapping Populations

Three different HF *R* genes (*H6*, *Hdic,* and *H5*), each in a different line of wheat, were examined in this investigation. Each wheat is maintained in the USDA-ARS Hessian fly laboratory at Purdue University. *H6* was discovered in bread wheat (*T. aestivum*) and is homozygous in the soft red winter wheat cultivar Caldwell (Patterson et al., 1982). Caldwell was developed, in part, to resist HF infestation in the Eastern United States. Caldwell seedlings were used to identify *H6*-virulent HF males as described below. *Hdic* was discovered in emmer wheat (*T. turgidum* ssp. *dicoccum*, PI 94641) and transferred to bread wheat, *T. aestivum* (Brown-Guedira et al., 2005). The homozygous *Hdic* hard winter wheat (KS99WGRC42) that was used to map *Hdic* in wheat (Liu et al., 2005a) was used to identify *Hdic*-virulent HF males in this investigation. *H5* was discovered in the Portuguese spring wheat cultivar Ribeiro (Shands and Cartwright, 1953). *H5* was backcrossed into the soft red winter wheat to produce the cultivar Abe (Patterson et al., 1975). Abe seedlings were used to identify *H5*-virulent males in a previous study (Behura et al., 2004). DNA extracted from some of those insects was used in the present investigation.

**Figure 1:**
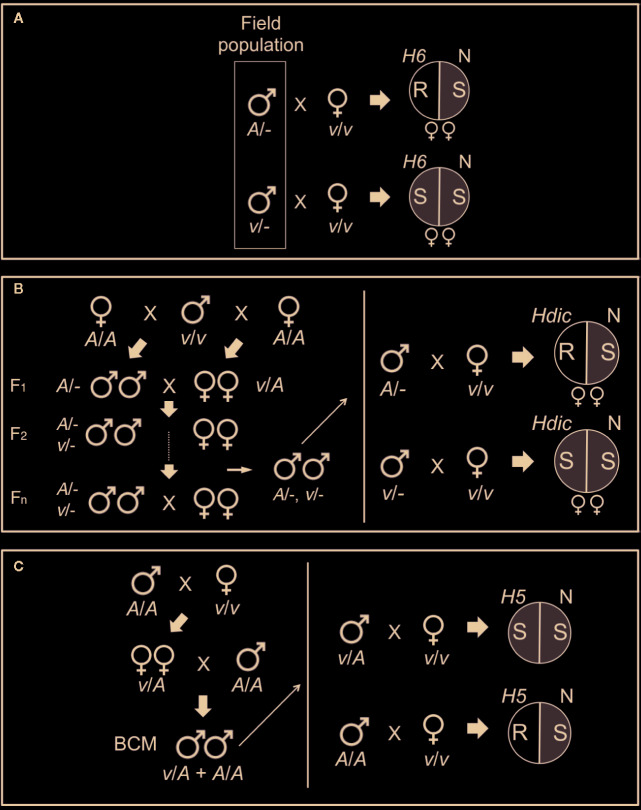
Genotyping HF mapping populations. **(A)** Males collected from a Louisiana field population (boxed) were individually mated with single, homozygous *H6*-virulent (*v/v*), virgin females that produced offspring of only one sex. After mating, the females were placed separately in pots containing *H6*- and Newton (N) seedlings and allowed to oviposit on the plants. The eggs were allowed to hatch and the larvae were allowed to feed on the plants. Avirulent parental males (*A/-*) produced avirulent female (*v/A*) larvae incapable of galling *H6*-plants (R, plant resistance). Virulent parental males (*v/-*) produced virulent female (*v/v*) larvae capable of galling *H6*-plants (S, plant susceptibility). The sex of the offspring was determined by allowing larvae to develop into adults on the Newton seedlings in each pot. Matings that produced only male offspring were uninformative, because males carry only their mother’s X chromosome. **(B)** An advanced intercross population (AIP) segregating for *Hdic* virulence and avirulence was initiated with a cross between a single *Hdic*-virulent (*v/v*) male and two sister avirulent (*A/A*) females (left panel). One female produced only female offspring and the other produced only male offspring. Males and females in the F_1_ and subsequent generations developed and were allowed to inter-mate and reproduce on susceptible plants. Males selected after the F_1_ generation were genotyped individually as described in A where *Hdic* indicates *Hdic*-resistant plants (right panel). **(C)** An *H5* virulence mapping population was initiated from a single mating between a homozygous *H5*-virulent (*v/v*) female and a homozygous *H5*-avirulent (*A/A*) male (left panel). F_1_ females developed on susceptible plants and were backcrossed to a single homozygous *H5*-avirulent (*A/A*) male. Backcross male offspring (BCM) were allowed to develop on susceptible plants and then selected for genotyping (right panel). Heterozygous (*v/A*) males mated to homozygous virulent (*v/v*) females produce two types of offspring: heterozygous (*v/A*) avirulent offspring and homozygous virulent (*v/v*) offspring capable of galling *H5*-seedlings (S). Homozygous (*A/A*) males produce only heterozygous (*v/A*) avirulent offspring incapable of galling *H5*-seedlings (R).

To map *H6* virulence, association mapping was performed using a non-structured Louisiana field population in which both virulence and avirulence to *H6*-wheat had been detected (Garcés-Carrera et al., 2014). Individual males in the Louisiana population were genotyped for *H6* virulence in separate testcrosses with homozygous *H6*-virulent virgin biotype-L females (**Figure 1A**). As described previously (Stuart et al., 1998), the ability of the offspring of each testcrossed male to gall and survive on *H6*-resistant (Caldwell) wheat seedlings determined male genotype. Genotyped males were collected, and their DNA was extracted using the DNeasy tissue kit (Qiagen, Chatsworth, CA).

To map *Hdic* virulence, we first isolated an *Hdic*-avirulent strain and an *Hdic*-virulent strain from an Israeli HF population. The *Hdic*-avirulent strain was selected using previously described methods (Zhao et al., 2015). Briefly, single mated females were caged and allowed to lay eggs on wheat seedlings in caged split-pots. One side of the pot contained susceptible Newton seedlings and the other side of the pot contained *Hdic*-resistant seedlings. Ten days after egg deposition, pots with *Hdic*-seedlings containing galled plants or living larvae were discarded. The larvae on susceptible (Newton) seedlings in the pots containing resistant *Hdic*-plants were allowed to develop. The emerging adult males and females were then intermated. This procedure was repeated for two additional generations, at which point no *Hdic* virulence was detected in the population. The *Hdic*-virulent strain was selected according to the method of Zantoko and Shukle (1997). For three generations, individual larvae were selected, one larva per plant, for the ability to survive, and gall *Hdic*-resistant seedlings. Surviving adults were collected and intermated to produce the *Hdic*-virulent strain.

The *Hdic* virulence mapping population was created by crossing a single virulent male with two avirulent sister females (one male-producing and one female-producing; **Figure 1B**). The resulting F1 male and female offspring were subsequently intercrossed to generate a *Hdic*-virulent advanced interbred population (vHdic-AIP). To maintain this population, individuals within vHdic-AIP were allowed to intermate and reproduce on *Hdic*-wheat. This process also served to disrupt linkage disequilibrium in the population. Individual F_2_, F_6_, and F_10_ males were genotyped as hemizygous *Hdic*-virulent (v/-) or *Hdic*-avirulent (A/-) in testcrosses with individual, homozygous, *Hdic*-virulent (v/v), virgin females (**Figure 1B**). Genotyped males were used for genomic DNA extraction and samples were pooled as described below.

The *H5* virulence mapping population (vH5-BCM) was developed previously (Behura et al., 2004). Briefly, *H5*-avirulent males (Great Plains biotype; GP) and *H5*-virulent females (biotype L) were intermated and F_1_ female offspring were backcrossed to a GP male to obtain vH5-BCM male offspring (**Figure 1C**). Since Hessian fly males transmit only their maternally derived chromosomes, the vH5-BCM males were testcrossed to homozygous, *H5*-virulent, biotype-L females, and their genotypes were determined by scoring the ability of their offspring to gall and survive on *H5*-resistant wheat (Abe) seedlings. Genotyped vH5-BCM males (n = 102) were collected for genomic DNA extraction. Behura et al. (2004) used DNA extracted from each of these males separately to map *H5* virulence to HF chromosome A2 (**Table 1**). DNA extracted from 48 of these males was used in the present investigation.

**Table 1:** HF *Avr* gene mapping approaches and results in this and previous investigations.

*R* ^a^	Approach^b^	Population (n)^c^	Chrom.^d^	Res^e^	No.^f^	Reference
*H3*	BSA, AFLP	BC_1_ (68)	A2	Un	0i	Behura et al., 2004
*H5*	BSA, AFLP	BC_1_ (102)	A2	Un	0i	Behura et al., 2004
*H5*	BSA-seq	BC_1_ (48)*	A2q	1.3	26i	This investigation
*H6*	BSA, M, B	F_2_-F_8_ (335)	X2q, m	0.3	2i/1p	Zhao et al., 2015
*H6*	BSA-seq	NS (52)	X2q, m	6.1	1f	This investigation
*H9*	BSA, M, B	F_2_-F_6_ (274) NS (92)	X1p, t	0.02	2i/1p	Zhao et al., 2015
*H13*	BSA, CW, M, B	F_2_ (223) NS (79)	X2p, t	0.02	2i/1c/1f	Aggarwal et al., 2014
*H24*	BSA, M	F_2_ (77) F_4_ (66)	X1q, t	0.24	4i/1p	Zhao et al., 2016
*Hdic*	BSA-seq	F_10_ (48)	X2q, c	2.1	Un	This investigation
*Hdic*	M	F_10_ (48)**	X2q, c	1.1	8i/2p	This investigation

^a^Wheat R gene against which HF virulence was mapped. ^b^Mapping approach, where BSA is bulked-segregant analysis, AFLP is amplified fragment length polymorphism, BSA-seq is BSA coupled with whole genome resequencing, M is microsatellite markers, B is bacterial artificial chromosome sequencing and CW is chromosome walking. ^c^Mapping populations where BC_1_ is a laboratory-produced backcross male population, F_n_ is a laboratory recombinant inbred male population selected at the n generation and NS is one or more field-derived non-structured populations. * is a subpopulation of that used by Behura et al. (2004). ** is the same population used in the Hdic BSA-seq experiment. ^d^Chromosome position where A2 is autosome 2, X1 is X chromosome 1, X2 is X chromosome 2, q indicates long arm, p indicates short arm, m is the middle of the chromosome arm, t is telomeric, and c is centromeric. ^e^Genomic resolution is the distance (Mb) between the closest flanking recombinant markers or the distance where the Fisher’s exact test -Log10(p-value) is greater than 1.7 (H6 BSA-seq, **Figures 3**) or greater than 5.8 (H5 BSA-seq and Hdic BSA-seq, **Figures 3** and **4**). ^f^Number of signal peptide-encoding genes identified (i) within the resolved region; the number of candidate Avr genes proposed (p) based on the presence and absence of transcription in avirulent and virulent larvae; the number of Avr genes confirmed (c) using RNA-interference gene silencing, and the number of genes showing effector activities in the functional assays performed in the present investigation (f). (Un, undetermined).

#### Sample Pooling and Genome DNA Sequencing

DNA bulks were prepared by mixing approximately equal amounts of genomic DNA from each male used in the study. Paired-end (PE) sequencing libraries were prepared (100 bp PE reads, ~250bp insertion size) and genomic DNA sequencing (Illumina HiSeq2000) was performed by the Purdue Genomics Core Facility (Purdue University, West Lafayette, Indiana, USA; **Table S1**). The PE reads were later trimmed with Trimmomatic (v.0.3.2) (Bolger et al., 2014) to remove adapters (settings: ILLUMINACLIP : TruSeq3-PE.fa:2:30:10:2:keepBothReads LEADING:3 TRAILING:3 MINLEN:50) and filtered for quality (Phred quality ≥Q20) with FASTX-toolkit (v.0.7) (settings: fastq_quality_filter -q 20 -p 80) (http://hannonlab.cshl.edu/fastx_toolkit/index.html).

#### Read Mapping and SNP Analysis

The pre-processed and quality filtered Illumina PE reads from each bulked DNA sample were mapped to the HF reference genome (GenBank assembly accession number GCA_000149185.1) using BWA v. 0.7.5a commands aln and sampe with default settings (Li and Durbin, 2010). SAMtools v.0.1.18 (Li et al., 2009) was used to remove ambiguously mapped and duplicated reads, keeping only those with a mapping quality higher than Q20 and proper mapped pairs. The SAMtools mpileup command was used to build a multiple-pileup file for SNP calling. SNPs around indels in the HF reference genome were filtered using the Perl scripts identify-indel-regions.pl (–indel-window = 5; window of 5bp in both directions) and filter-sync-by-gtf.pl. The final filtered mpileup file was synchronized using the java tool mpileup2sync.jar, filtering for base quality higher than Q20. SNP allele frequencies were estimated using the Perl script snp-frequency-diff.pl for bi-allelic SNPs using the following settings: –min-count = 4 (the minimum read count of the minor allele considering all bulks simultaneously); –min-coverage = 10 (the minimum read coverage per bulk used for SNP identification); and –max-coverage = 200 (the maximum read coverage per bulk used for SNP identification). These criteria were used in order to reduce the possibility of predicting false SNPs in genomic regions with poor sequencing coverage or repetitive DNA sequences. The statistical significance of allele frequency differences for each SNP position was determined with Fisher’s exact test (FET) using Perl script fisher-test.pl. Fixation index (FST) values were determined for each SNP with Perl script fst-sliding.pl. The java tool mpileup2sync.jar and other Perl scripts used for SNP filtering and statistical analyses are included in the Popoolation2 tool (Kofler et al., 2011). The IGV genome viewer (Thorvaldsdóttir et al., 2013) was used to visualize the mapped reads as well as the FET and FST analyses. The average FET and FST values for 10-kb sliding-windows (5-kb steps) were plotted using R programming language [plot() function] as the cubic-smoothed line [smooth.spline() function] in order to reduce noise from sequencing variation across the HF genome. The Bonferroni correction method was used to establish the genome-wide statistical cutoff for FET analyses. Using an α value of 0.05 and 31,600 10-kb genome windows across the 158-Mb HF genome established an FET significance cutoff value of 1.58e-6 (-Log10[FET] = 5.8).

#### Data Availability

Whole-genome sequencing data for bulked samples are available at the NCBI Sequence Read Archive (SRA) under the NCBI Bioproject accession number PRJNA613640 (https://www.ncbi.nlm.nih.gov/bioproject/?term=PRJNA613640).

### Genetic Mapping With PCR-Based Markers

The Hessian fly reference genome was used to map genes and identify PCR-based (microsatellite) markers and design PCR primers with the SSR Locator software (da Maia et al., 2008). The gene model identifiers are the names in the official HF gene set (OGS). These can be accessed at the USDA Arthropod i5k official workspace https://i5k.nal.usda.gov/data/Arthropoda/maydes-(Mayetiola_destructor)/GCA_000149185.1/ and in the genome assembly curated at the National Center for Biotechnology Information (NCBI), GenBank assembly accession number GCA_000149185.1. Molecular markers were used to genotype individuals taken from mapping populations and pooled DNA samples using standard PCR methods and the primers listed in **Table S2**.

### Fluorescent In Situ Hybridization (FISH)

The end-sequences of HF genomic bacterial artificial chromosomes (BACs) that had been mapped to HF polytene chromosomes (Aggarwal et al., 2009) were used as part of the HF genome sequencing project (Zhao et al., 2015). We used these data to identify HF BACs that reside within HF genome scaffolds A1Random.66 and X2.8. From among these BACs, we selected BAC HF07L11 as a probe for scaffold X2.8 and BAC Md23L24 as a probe for scaffold A1R.66. Using methodology described previously (Stuart et al., 2014), these BAC clones were fluorescently labeled, denatured, and allowed to hybridize to complementary bases on HF polytene chromosome preparations. Later, the chromosomes were stained with DAPI and the positions of BAC hybridizations were examined and photographed using fluorescence microscopy.

### Reverse Transcription PCR Analyses

To perform reverse transcription PCR (RT-PCR), total RNA was isolated from pools of 50 to 60 two-day-old first-instar larvae. RNA from avirulent and virulent strains were extracted separately using the RNeasy Mini Kit (Qiagen). Single-strand cDNA was reverse transcribed using the RNA of each individual pool separately and the SuperScript III First Strand kit (Invitrogen) according to the manufacturer’s recommendations. Single-strand cDNA was then used in PCR experiments using gene-specific primers (**Table S3**). PCR was performed in 35 cycles of 95°C for 30 s, 55°C for 30 s, and 72°C for 60 s, followed with a final step of 72˚C for 5 min. The Hessian fly actin gene was included as a reference and internal control (**Table S3**). RT-PCR products were visualized on agarose gels. RT-PCR amplifications were performed with at least three biological replications.

### Phyre2 Structural Protein Modeling

Phyre2 (http://www.sbg.bio.ic.ac.uk/~phyre2/) is a free web-based service for prediction of the three-dimensional (3D) structure of a protein sequence using homology modeling against a database of Hidden Markov Model (HMM) profiles of known 3D protein domain structures from the Protein Data Bank (PDB, http://www.wwpdb.org/) (Kelley et al., 2015). Phyre2 was used to examine the predicted protein structures of the two best candidate *vHdic* genes in an attempt to identify similarities with the domains of other proteins.

### Candidate HF Effector Gene Cloning and Plasmid Construct Preparation

Total RNA was isolated from HF, first-instar, larval biotype GP using the RNeasy Mini Kit (Qiagen). RNA was subsequently reverse transcribed into first-strand cDNA using the SuperScript III First Strand Synthesis kit (Invitrogen). Double-stranded cDNA for effector genes was amplified from first-strand cDNA using gene-specific primers containing Gateway attB adapters (**Table S4**). These primers were designed to exclude the corresponding secretion signal peptides from each effector gene. Gene attB PCR products were recombined into pENTR/pDONR vectors using the Gateway BP reaction (Invitrogen) and chemically transformed into *Escherichia coli* OmniMAX2 cells (Invitrogen). Recombinant colonies were selected on 50 µg/ml kanamycin LB plates. Colonies carrying the recombinant plasmids were selected for plasmid isolation and DNA insert sequencing. Genes in pENTR/pDONR vectors were recombined by Gateway LR reactions (Invitrogen) into expression vector pEDV6 and transformed into *E. coli* OmniMAX2 cells. Recombinant colonies were selected on 10 µg/ml gentamicin LB plates and used for plasmid isolation. A pEDV-GFP construct was built by LR recombination between plasmids pENTR1AGFP-N2 (FR1) (Campeau et al., 2009) and pEDV6 (Sohn et al., 2014). Plasmid pEDV5 (Sohn et al., 2007) was used as an empty vector (EV) control. pENTR1A-GFP-N2 (FR1) was a gift from Eric Campeau (Addgene plasmid 19364). Plasmids pEDV5/6 were generously supplied by J. Jones (The Sainsbury Laboratory, Norwich U.K.).

### Mobilization of pEDV Constructs Into Bacteria

The pEDV constructs were transformed into *B. glumae* cells as follows: Electrocompetent *B. glumae* cells were prepared as previously described (Saitoh and Terauchi, 2013) with minor modifications. In brief, the *B. glumae* strain 336gr-1 was inoculated in 20 ml LB medium for 14-16 h at 28°C with shaking until OD600 = 0.8. The flask was then opened for 30 s under clean conditions and then maintained at 28°C with shaking for an additional 4 h. The cells were then pelleted twice at 4°C and 3000 rpm for 5 min and resuspended in cold 10% glycerol. The pellet was then dissolved in 600 μl of cold 10% glycerol and divided into 50-μl aliquots. These were stored at -80°C for transformations. Each plasmid construct (0.3 μg) was electroporated into *B. glumae* using a MicroPulse Electroporator (Bio-Rad). Transformant *B. glumae* strains were selected on gentamicin (25 µg/ml) LB agar. *B. glumae* strain 336gr-1 was a generous gift from J. H. Ham (Dept. Plant Pathology and Crop Physiology, Louisiana State University).

### Hypersensitivity Reaction (HR) Induction/Suppression Assays

For HR assays, *Bglu*-pEDV strains were plated on King’s Broth (KB) agar 25 µg/ml gentamicin and incubated at 30°C for 14 to 16 h. *Bglu*-pEDV strains were dissolved in 0.9% NaCl solution at OD600 = 0.7. Bacteria suspensions were infiltrated with a 1-ml syringe without a needle into 4- to 5-week-old *Nicotiana tabacum* Burley 21 HA and *N. benthamiana* leaves. Infiltrated plants were maintained in a growth chamber at 24 ± 1°C with a 16:8 (light/dark) light cycle and 80 ± 5% relative humidity. HR was recorded after 24 h for *N. tabacum* and 48 h for *N. benthamiana*.

### Ion-leakage Assays

*Bglu*-pEDV cell suspensions were prepared and infiltrated in leaves of *N. tabacum* and *N. benthamiana* plants as described above. Leaf disks (150 mm diameter) were collected from the infiltrated areas using a cork borer 18-h post infiltration (hpi) for *N. tabacum* and 36 hpi for *N. benthamiana*. Leaf disks were floated on 15-ml nanopure water and incubated at 22°C with gentle shaking (100 rpm). Conductivity in the water was registered after 4 hours of incubation using a conductivity meter (Metler Toledo S30K) with a sensor probe (Conductivity Sensor LE703, Metler Toledo). Samples from three different plants were used as replicates for each treatment. Statistical analyses were performed using ANOVA and Tukey’s HSD for significant differences (p < 0.05) among treatments.

## Results

### BSA-Seq Confirms the Genomic Position of *H6* Virulence

**Figure 2:**
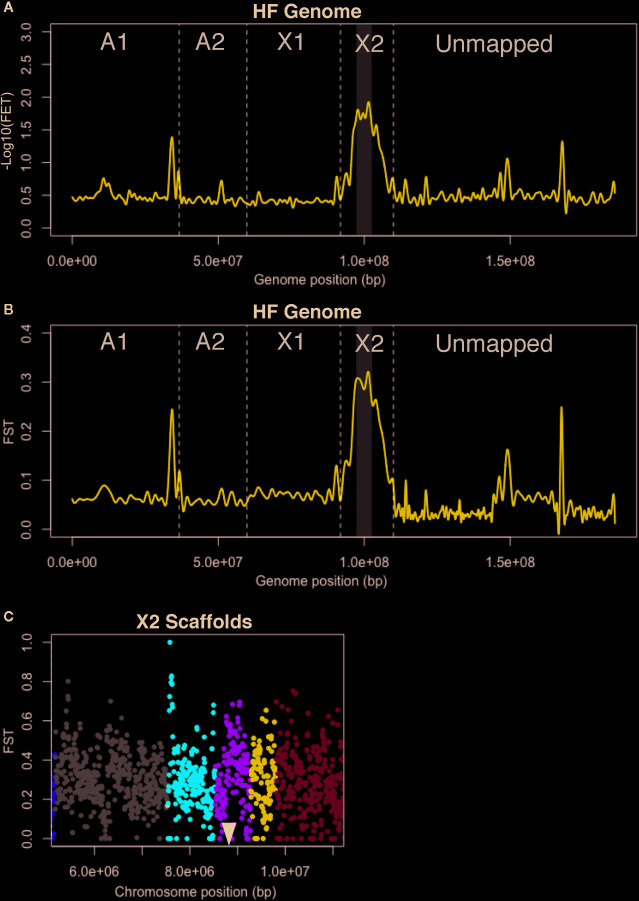
*H6* virulence BSA-seq analysis. **(A)** Fisher’s exact test (FET) for significance of SNP allele frequency differences between *H6*-avirulent and *H6*-virulent bulks plotted as a cubic-smoothed line. Average -Log10(FET) values for 10-kb sliding-windows (5-kb steps) were plotted across the HF genome. Vertical dashed lines separate scaffolds assigned to HF chromosomes (A1, A2, X1, and X2) and unassigned scaffolds (Unmapped). Gray shading indicates the genomic position of a 6.1-Mb chromosome-X2 region where -Log10(FET) is less than the 5.8 statistical cutoff, but greater than 1.7. **(B)** FST values for 10-kb sliding-windows (5-kb steps) plotted as a cubic-smoothed line across the HF genome. The 6.1-Mb region highlighted in A has the highest FST values (gray shading). **(C)** FST estimates within the 6.1-Mb chromosome-X2 region highlighted in B. Each dot represents the average FST values for 10-kb sliding-windows (5-kb steps). The scaffolds in this region are shown with dots of different colors; X2.8 is grey, X2.10 is red, X2.11 is green, X2.12 is dark blue and X2.13 is light blue. A black triangle indicates the position of the previously cloned candidate-*vH6* gene, Mdes009086-RA (Zhao et al., 2015).

Zhao et al. (2015) mapped *H6* virulence on the long arm of HF chromosome X2 using four different structured mapping populations and PCR-based DNA markers (**Table 1**). This had been an arduous task. Therefore, we decided to test the capacity of BSA-seq in the HF using *H6* virulence. *H6*-virulent bulk DNA was prepared from 23 males. *H6*-avirulent bulk was prepared from 19 males. Each male was collected from a field-derived population and genotyped in individual testcrosses (**Figure 1A**). These bulks were sequenced separately, resulting in 5.32 Gb of combined genomic data (**Table S1**). The BWA tool (Li and Durbin, 2010) aligned the reads from each bulk against the HF reference genome and then SAMtools (Li et al., 2009) used these mapping data to identify 1.5 million SNPs. Popoolation2 (Kofler et al., 2011) calculated SNP allele frequencies within each bulk and then determined the differences in allele frequency for each SNP. Using these data, Fisher’s exact test (FET) was performed and average -Log10(FET) values within sliding 10-kb genome windows were plotted as a smoothed line across the HF genome (**Figure 2A**).

Unfortunately, the HF genome sequence is imperfectly assembled, and this was evident in the plotted data. Instead of a single peak that rose and fell over a single chromosomal position, several peaks were observed scattered along the genome map in both FET and FST plots (**Figure 2A, B**). These peaks indicate that linkage exists between *H6* virulence and the underlying genome scaffolds. Most of these scaffolds are located in the “unmapped” fraction of the genome map. Their peaks suggest that they should be assigned to the chromosome known to carry the *H6* virulence trait (Zhao et al., 2015), chromosome X2. Unmapped scaffold Un.18557, with an associated -Log10[FET] of 1.3, was the clearest example. Another peak with the same elevation was associated with a single chromosome A1 scaffold (A1R.66). The same logic suggests that all or part of scaffold A1R.66 also belongs to chromosome X2. This was later confirmed when *Hdic* virulence was mapped (described below).

Importantly, the peak with the greatest elevation (-Log10[FET] = 1.7) identified a 6.1-Mb region on the long arm of chromosome X2 (**Figure 2A**). Although this value failed to meet the statistical cutoff, established using the Bonferroni correction method (-Log10[FET] = 5.8), the 6.1-Mb genome region under this peak includes the 300-kb genome window where *H6* virulence was previously mapped (Zhao et al., 2015). This region contains HF genome scaffolds X2.8, X2.10, X2.11, X2.12, and X2.13 (**Figure 2C**). Using Web Apollo (Kofler et al., 2011; Lee et al., 2013) and the HF genome reference sequence (https://i5k.nal.usda.gov/Mayetiola destructor) we identified 945 gene models within this window. Gene model Mdes009086-RA, which was previously identified as the candidate gene conditioning *H6* virulence (*vH6*), resides within scaffold X2.11 (**Figure 2C**). Mdes009086-RA encodes a member of the putative HF effector protein family SSGP71. It is not transcribed in *H6*-virulent larvae (Zhao et al., 2015).

Although this investigation failed to resolve the position of *H6* virulence as well as the previous investigation (**Table 1**), the results demonstrated that non-structured field-derived populations can be used to identify SNPs linked to HF virulence and encouraged us to use BSA-seq to try to resolve the positions of virulence to other HF *R* genes. As discussed below, we believe that a larger mapping population would have improved *H6* virulence mapping resolution.

### BSA-Seq Resolves *vHdic* Position and Corrects the HF Genomic Map

To select a HF strain that was *Hdic*-virulent, the offspring of individual females taken from an Israeli field collection were examined for their ability to survive and stunt *Hdic*-wheat. During this process, we noted that all matings (n = 9) between individual *Hdic*-virulent females and individual *Hdic*-avirulent males produced *Hdic*-virulent male offspring. This suggested that *Hdic* virulence is X-linked because male offspring do not inherit X chromosomes from their fathers; matings between virulent females (*v/v*) and avirulent males (*A/-*) produce only virulent male offspring (*v/-*).

To test this possibility, we looked for linkage between X-linked microsatellite markers using conventional BSA. F_2_ males collected from the *Hdic* virulence advanced intercross population (vHdic-AIP) were genotyped as *Hdic*-avirulent or *Hdic*-virulent individuals (**Figure 1B**). Separate avirulent and virulent DNA bulks were then prepared and used as template with primers designed for those microsatellites in separate PCR experiments. The two pools amplified alternative microsatellite alleles with six markers on scaffolds X2.7 and X2.8 (data not shown), indicating that those markers were linked to *Hdic* virulence. Genetic mapping using X2.7 and X2.8 markers and the DNA of 189 individual males collected from the F_2_, F_6_, and F_10_ generations confirmed this linkage and suggested that *Hdic* virulence was present on either a 100-kb fragment on the proximal end of scaffold X2.8 or within the gap between X2.7 and X2.8.

**Figure 3:**
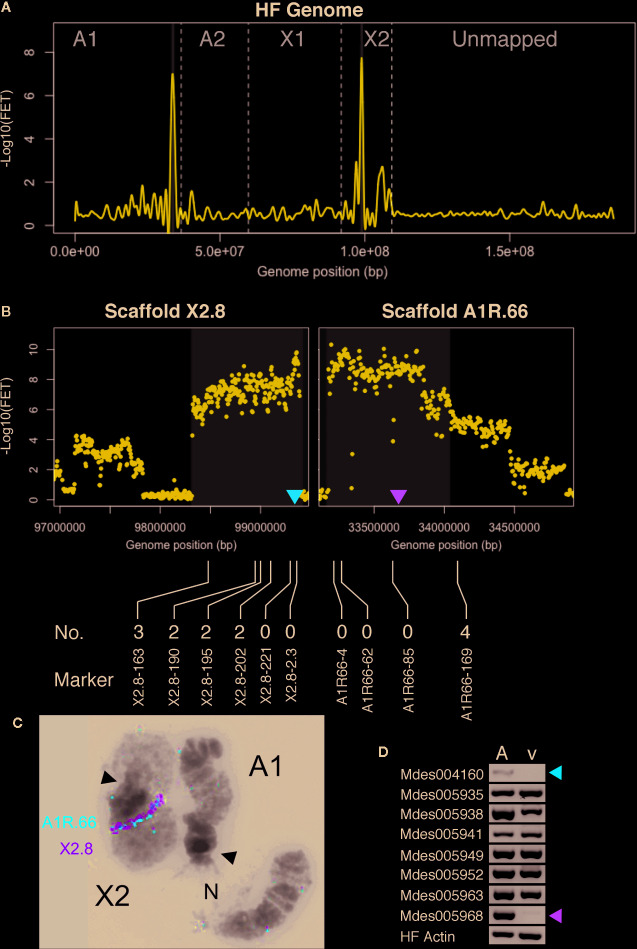
*Hdic* virulence BSA-seq analysis. **(A)** Fisher’s exact test (FET) analysis for significance of SNP allele frequency difference between *Hdic*-avirulent and *Hdic*-virulent bulks. The average -Log10(FET) values are plotted and the relative positions of scaffolds on chromosomes are presented as in **Figure 2A**. Genomic regions where -Log10(FET) values are statistically significant (> 5.8) are highlighted in gray. These regions correspond to sequences within scaffolds A1R.66 and X2.8. **(B)** FET analysis of scaffolds X2.8 and A1R.66. Each dot represents average FET values for 10-kb sliding-windows (5-kb steps). Sequences where -Log10(FET) values are statistically significant (> 5.8) are highlighted. Red and green triangles indicate the genomic positions of candidate genes Mdes004160 and Mdes005968, respectively. The positions of X2.8 and A1R.66 markers used to refine the genomic map are shown below the plots. The number of *Hdic*-virulent recombinant individuals (out of 48 total) is shown directly above each marker’s designation. **(C)** Fluorescence *in situ* hybridization (FISH) of BAC-based probes to HF polytene chromosomes X2 and A1. Scaffold X2.8 BAC HF07L11 (green signal) and scaffold A1R.66 BAC Md23L24 (red signal) hybridized adjacent to each other on chromosome X2. White arrows indicate chromosome centromeres (N = nucleolus). **(D)** RT-PCR using total RNA isolated from *Hdic*-avirulent (A) and -virulent (v) first instar HF larvae. Gene-specific primers for all eight putative signal-peptide encoding genes amplified *Hdic*-avirulent cDNA. The Mdes004160 and Mdes005968 primers failed to amplify *Hdic*-virulent cDNA. The HF actin gene was used as a positive control. Similar results were obtained in each of three independent biological replications.

For BSA-seq analysis, an *Hdic*-avirulent DNA bulk (n = 15) and an *Hdic*-virulent DNA bulk (n = 33) were prepared using DNA extracted from F_10_ males (**Figure 1B**). Each bulk was separately sequenced. This produced 12.2 Gb of combined genomic data containing 1.2 million SNPs (**Table S1**). The average FET values for SNPs across sliding 10-kb genome windows were plotted (**Figure 3A**). Two genomic regions were associated with peaks where -Log10(FET) values were greater than the statistical cutoff of 5.8. These appeared to be tightly linked to *Hdic* virulence. Consistent with the conventional BSA results, one region included a 1.1-Mb section of scaffold X2.8 (**Figure 3B**). Unexpectedly, the other region contained a 1.0-Mb section of scaffold A1R.66, the same A1 scaffold that showed X2 linkage in the *H6* virulence investigation described above.

We hypothesized that A1R.66 was contiguous with scaffold X2.8, and present in the gap between scaffolds X2.8 and X2.7 (**Figure S1**). That hypothesis was tested *via* genetic mapping using PCR-based A1R.66 markers (**Table S5**) and 48 individual vHdic-AIP F_10_ males. Consistent with the hypothesis, A1R.66 was linked to both X2 and *Hdic* virulence in those experiments (**Figure 3B**). It was tested again using fluorescence *in situ* hybridization (FISH) to the HF polytene chromosomes. An X2.8 BAC and an A1R.66 BAC used as probes hybridized together on the long arm of chromosome X2 (**Figure 3C**). Taken together, the genetic and FISH data determined that the most likely order of the scaffolds on the long arm of chromosome X2 is centromere-X2.7-A1R.66-X2.8. Therefore, disregarding the gap between scaffolds X2.8 and A1R.66, BSA-seq data positioned *Hdic* virulence within a contiguous 2.1-Mb region on the long arm of chromosome X2 (**Table 1**, **Figure 3B**). The PCR markers used in this investigation further resolved the *Hdic* virulence, to within a 1.1-Mb region flanked by the closest recombinant markers, X2.8-202 and A1R66-169 (**Figure 3B** and **Table 1**).

#### Candidate *vHdic* Genes Identified

Using Web Apollo (Lee et al., 2013) and the HF genome reference sequence (https://i5k.nal.usda.gov/Mayetiola_destructor), 48 gene models on A1R.66 and six gene models on X2.8 were identified within the 1.1-Mb region identified above. The SignalP4.1 algorithm (Petersen et al., 2011) predicted that eight of these genes models encode proteins containing secretion signals (**Table S6**). Three of these had significant sequence similarities to other genes in insects (BLASTP, e ≤ 3e-87). Five others were predicted HF effector proteins. Four of these belong to gene family SSGP4, which consists of at least 64 predicted HF effector-encoding genes (Zhao et al., 2015). SSGP4-encoded proteins that have no sequence similarities to any other proteins thus far identified outside of the HF (BLASTP, e > 5.0).

Using RT-PCR, we examined whether each of the eight signal-peptide-encoding genes is transcribed in first instar larvae. Transcripts of all eight genes were detected in *Hdic*-avirulent larvae (**Figure 3D**). However, transcripts of two SSGP4 genes, Mdes004160-RA and Mdes005968-RA, were not detected in *Hdic*-avirulent first-instar larvae. Because *Avr* gene loss-of-function often correlates with virulence, these observations make Mdes004160-RA and Mdes005968-RA good candidate *vHdic* genes. The predicted protein sequences of these genes are presented in **Figure S2**. Although their secretion-signal peptides are 75% identical, their mature proteins are only 32% identical.

Previous investigations have used Phyre2 (Kelley et al., 2015) to identify putative protein domains in predicted HF effectors (Zhao et al., 2015; Zhao et al., 2016). Therefore, we used Phyre2 to predict the 3D structures of the Mdes005968-RA- and Mdes004160-RA-encoded proteins in an attempt to identify similarities with known protein domain structures. This analysis failed to identify any protein with significant structural similarities with either predicted protein (e-value ≥ 1). Because it is located in the center of the *vHdic*-mapping window (**Figure 3D**), we selected gene Mdes004160 for the functional analysis described below.

### BSA-Seq Maps *H5* Virulence to HF Genomic Scaffold A2.7

Previously, *H5* virulence was mapped to a region spanning the centromere of chromosome A2. That region composed 30% of the chromosome’s length (Behura et al., 2004), and in three independent experiments, displayed severe recombination suppression. Therefore, in the present investigation, we first examined microsatellite markers identified on scaffolds flanking the A2 centromere for linkage to *H5* virulence. To do this, we used the DNA extracted from 36 F_2_ back-crossed males genotyped in the Behura et al. (2004) study (**Figure 1C**). The DNA of each male was examined separately for each marker. Consistent with the recombination suppression previously observed, each scaffold examined was linked to *H5* virulence (**Table S7**). However, recombination was completely lacking between *H5* virulence and markers on scaffolds A2.6 (458.5 kb) and A2.7 (1.3 Mb), suggesting that the resolution of the position of *H5* virulence might improve with a larger mapping population and additional markers.

In an attempt to do this, BSA-seq was applied to DNA bulks from 24 additional *H5*-avirulent and 24 additional *H5*-virulent (n = 24) males selected from the same F_2_ back-crossed mapping population (**Figure 1C**). Because *H5* virulence is autosomal, the *H5*-virulent bulk was developed from heterozygous (*v/A*) males while the *H5*-avirulent bulk was developed from homozygous (*A/A*) males. Therefore, SNPs in the *H5*-avirulent bulk were expected to be heterozygous in genomic regions unlinked to *H5* virulence and homozygous in genomic regions linked to *H5* virulence.

**Figure 4:**
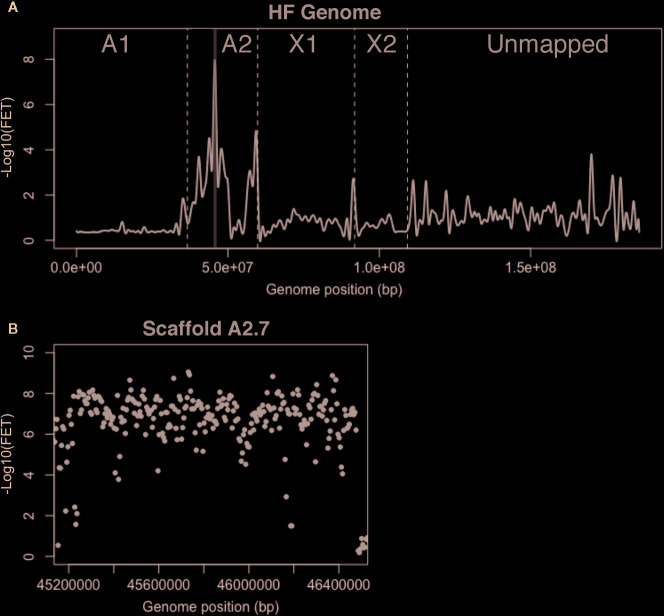
*H5* virulence BSA-seq analysis. **(A)** Fisher’s exact test (FET) analysis for significance of SNP allele frequency difference between *H5*-avirulent and *H5*-virulent bulks. The average -Log10(FET) values were plotted, and the relative positions of scaffolds on chromosomes are presented as in **Figure 2A**. A 1.3-Mb region corresponding to a single genome scaffold (A2.7) is highlighted in gray where the -Log10(FET) was statistically significant. **(B)** FET analysis of scaffold A2.7 (highlighted in panel **A**). Each dot represents the average -Log10(FET) value for 10-kb sliding-windows (5-kb steps).

Whole-genome sequencing produced 13-Gb of high-quality reads, containing about 0.92 million SNPs (**Table S1**). Average FET values for SNPs were plotted as described above (**Figure 4A**). Recombination suppression was again observed across much of the A2 chromosome as most A2 scaffolds had -Log10(FET) values of 2.0 and higher. In addition, one X1 scaffold and seven unmapped scaffolds had peaks with -Log10(FET) scores of 2.0 or greater, suggesting that they should probably be assigned to chromosome A2. Nevertheless, *H5* virulence appeared to be most tightly linked to scaffold A2.7, with a statistically significant peak -Log10(FET) value of 8.0 (**Figure 4A**). Plotting -Log10(FET) values for 10-kb sliding-windows across the scaffold revealed that SNPs across the scaffold had similar FET values (**Figure 4B**). Consequently, we suspected that *H5* virulence is probably associated with an *Avr* gene within the 1.3-Mb sequence of scaffold A2.7 (**Table 1**).

Scaffold A2.7 contained 142 gene models. Thirty-seven of these encode predicted proteins carrying secretion signal peptides. BLASTP indicated that 26 of these are highly conserved and unlikely effector candidates. The remaining 11 genes have no similarities with genes in other insects. We discovered that one gene model, Mdes007142-RA, was composed of two putative effector-encoding genes (**Figure S3A**); one called SSGP47-1 (Chen et al., 2008) and another referred to here as Mdes007142(b). We examined the transcription of all 12 of these genes using RT-PCR on first instar larvae in the *H5*-avirulent and *H5*-virulent strains. None of these were differentially transcribed between bulks (**Figure S3B**). Therefore, multiple alignment and manual comparisons were performed on DNA sequences of each bulk against each other and the reference sequence. These comparisons examined the sequences of the virulence- and avirulence-associated alleles of each gene for evidence of frameshift and nonsense mutations that might disrupt the proper translation of virulence-associated sequences. These comparisons failed to identify putative null mutations that might be associated with the virulence allele of a candidate *vH5* gene. Therefore, although BSA-seq was able to improve the resolution of *H5* virulence on chromosome A2, we were unable to associate *H5* virulence with a putative effector encoding gene. The possibility remains that a gene that has yet to be identified within the A2.7 sequence is responsible for *H5* virulence.

### Candidate-vH6 and vH13 Proteins Suppress Non-Host Plant Immunity

**Figure 5:**
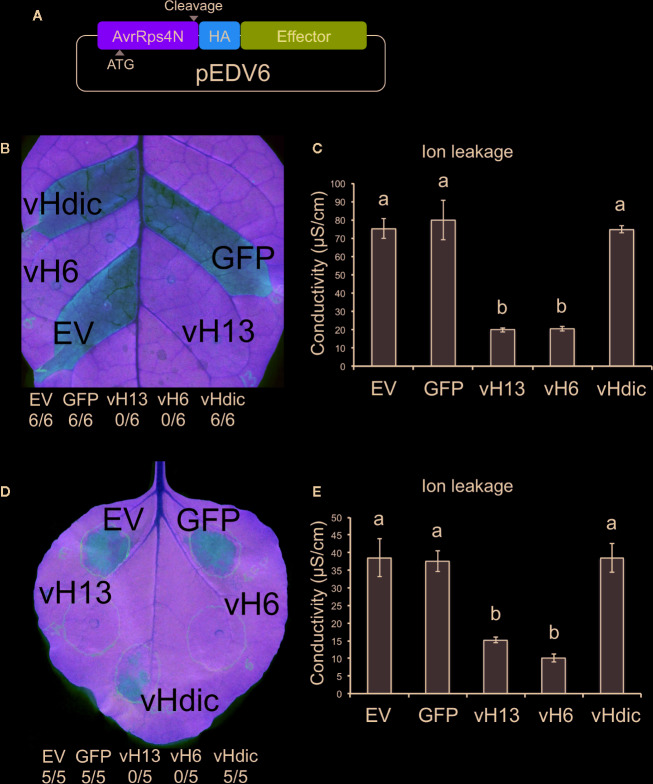
Reactions of *Nicotiana tabacum* and *N. benthamiana* to *Burkholderia glumae* carrying T3SS-fusion constructs for candidate *Mayetiola destructor* effector proteins. **(A)** Schematic representation of the pEDV6 constructs. AvrRps4N: N-terminal region (T3SS-secretion signal) of the bacterial AvrRPS4 protein; HA: hemagglutinin peptide (HA) tag; Effector: candidate effector gene for testing. Initiation codon (ATG) and cleavage site are indicated. **(B)** *N. tabacum* reaction to the infiltration of *B. glumae* harboring the pEDV constructs (Bglu-pEDV) for the avirulence *vH13* gene (vH13), avirulence *vH6* gene (vH6), candidate avirulence *vHdic* gene (vHdic, Mdes004160), the green fluorescent protein gene (GFP) and empty vector control (EV). The number of times each Bglu-pEDV strain induced HR in 6 replications is shown. Pictures were taken 48 hpi for both plant species. HR was visible at 24 hpi in *N. tabacum*. **(C)** Ion leakage assays of infiltrated leaf areas in *N. tabacum*, measured at 18 hpi. **(D)** *N. benthamiana* reaction to the infiltration of Bglu-pEDV strains for vH13, vH6, candidate vHdic, GFP, and EV. The number of times each Bglu-pEDV strain induced HR in 5 replications is shown. **(E)** Ion leakage assays of infiltrated leaf areas in *N. benthamiana*, measured at 18 hpi. For panels **(C, E)**, each bar represents the average conductivity from 3 independent plants. Error bars represent the standard error. Statistical differences among the treatments were found with ANOVA (*N. tabacum*: F = 31.59, p < 0.0001; *N. benthamiana*: F = 17.76, p = 0.0002) and Tukey’s test. Bars with different letters are significantly different (p < 0.05).

Based on the similarities that HF-wheat interaction has with pathogen-plant systems, we decided to explore the pEDV system for bacterial T3SS-dependent delivery of effector proteins into plant cells and test whether HF candidate effectors are capable of suppressing plant defense responses. As described above, candidate *vH6* (Mdes009086-RA) and one candidate *vHdic* (Mdes004160) were selected for this analysis. *vH13* was included as a third HF *Avr* gene (Aggarwal et al., 2014). Green fluorescent protein (GFP) was used as a negative control. Each gene was moved separately into the pEDV6 vector (**Figure 5A**) and each construct transformed into *B. glumae* for infiltration in non-host *N. tabacum* and *N. benthamiana* plants (Kang et al., 2008; Sharma et al., 2013). At 24 hpi in *N. tabacum* and 48 hpi in *N. benthamiana* (**Figures 5B, D**), the HR that *B. glumae* normally induces in *Nicotiana* was evident on leaf tissue infiltrated with *B. glumae* carrying an empty vector (EV), GFP and candidate *vHdic*. However, HR was reduced or absent on leaf tissue infiltrated with *B. glumae* harboring candidate *vH6* and *vH13*. These results were confirmed in a separate experiment in which ion leakage was used to assess plant cell integrity and membrane damage to the plant tissues (Rolny et al., 2011) (**Figures 5C, E**). Lack of HR suggests that, like many plant effectors, candidate-vH6 and vH13 proteins interfere with plant immunity.

#### Truncated vH6 Failed to Suppress Bacterial-Induced Plant Cell Death

**Figure 6:**
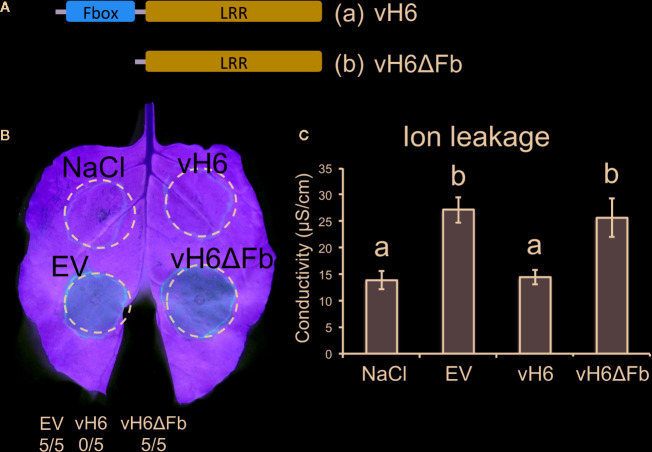
*Burkholderia glumae* carrying truncated *vH6* elicits HR-related cell death in *Nicotiana benthamiana*. **(A)** Schematic representation for (a) complete vH6 protein harboring the F-box domain (orange) and the leucine-rich repeats (LRR) domain (blue); and (b) truncated vH6 lacking the Fbox domain (vH6ΔFb). **(B)** *B. glumae* pEDV constructs (Bglu-pEDV) for complete vH6 (vH6), F-box-lacking vH6 (vH6ΔFb) and the empty vector control (EV) were infiltrated in leaves of *N. benthamiana*. Infiltration buffer (NaCl) was also included as a control. HR elicitation was recorded at 48 hpi. *B. glumae* carrying an empty pEDV vector (EV) elicits cell death. *B. glumae* carrying pEDV-vH6 fails to elicit cell death. Cell death is elicited when *B. glumae* carries truncated vH6 (vH6ΔFb). The number of times each strain elicited cell death in 5 replications is shown. **(C)** Ion leakage assay for infiltrated leaf areas of *N. benthamiana*. Bars represent the average conductivity measured at 18 hpi in 3 independent plants. Error bars represent the standard error. Statistical differences among the treatments were found with ANOVA (F = 9.05, p = 0.006) and Tukey’s test. Bars with different letters are significantly different (p < 0.05).

Candidate *vH6* is a member of a large family of HF effector-encoding genes (SSGP71). Like other SSGP71 proteins, candidate-vH6 contains both F-box and leucine-rich-repeat (LRR) domains (**Figure 6A**). The F-box domain of candidate-vH6 interacts with wheat Skp1-like proteins (Zhao et al., 2015), thereby mimicking a wheat E3 ligase. Therefore, we hypothesized that deleting the candidate-vH6 F-box domain from the effector would negatively impact its mode of action when delivered to *Nicotiana* cells by *B. glumae*. And as expected, unlike the complete candidate-vH6 effector (Bglu-vH6), the truncated candidate (Bglu-vH6ΔFb) did not interfere with nonhost HR on *N. benthamiana* in either the leaf or ion leakage assays (**Figures 6B, C**).

## Discussion

Virulence to seven different HF *R* genes in wheat has been positioned on HF chromosomes (**Table 1**). To our knowledge, outside of the HF, virulence to only one other plant *R* gene has been mapped, *Bph1* virulence in the brown planthopper (*Nilaparvata lugens*) (Kobayashi et al., 2014). In each case, BSA was used to identify linked DNA polymorphisms. Earlier HF investigations first sequenced BSA-discovered markers (*H5* and *H13*) and then, when it was feasible (*H13*), used the marker sequences to identify BACs that were later fully or partially sequenced themselves. The BAC sequence data was then used to develop new probes that permitted chromosome walking toward an *Avr* gene. PCR-based markers (microsatellites) identified in the sequence along the walk were then used to resolve the position of virulence until the virulence-associated mutations themselves were discovered. The development of a fully sequenced HF genome greatly simplified this process as chromosome walking and BAC sequencing were no longer necessary for microsatellite discovery. BSA-seq further simplifies the process as the DNA polymorphisms linked to virulence are discovered and positioned simultaneously.

Nevertheless, we found that conventional microsatellite mapping provided better resolution for virulence to both *H6* and *Hdic* than the BSA-seq performed here. However, because BSA-seq performance depends on estimations of SNP allele frequency within the bulked samples (Magwene et al., 2011; Rellstab et al., 2013), we believe that virulence resolution could be improved with DNA pools composed of greater numbers of individuals and better genome sequencing coverage, as larger sample sizes and higher-sequencing depth reduce the variability of SNP-allele frequency estimations and increase the chances of capturing recombination events.

In particular, the resolution of *H6* virulence was probably limited by the low genome-wide read coverage in relation to the bulk sizes (**Table S1**). Sequencing coverage is an important source of variation for allele frequency estimations because low coverage reduces the chance of capturing reads from each individual in the bulk. In general, genome sequencing coverage should be at least equal to the effective pool size (number of individuals multiplied by the ploidy level) in order to cover rare variants (Magwene et al., 2011; Rellstab et al., 2013). Because sequence coverage of the *H6*-avirulent and -virulent pools (14x and 14.6x) was less than each effective pool size (19 and 23), FET values were relatively low and linkage to *H6* virulence was relatively weak (FET p-value < 0.02). In comparison, *Hdic* sequence coverage (16.5x and 49.2x) was greater than each effective pool size (15 and 33) and linkage was much stronger (FET p-value < 1e-6). Although the *H5* effective pool sizes were higher (48 and 48, due to autosomal diploidy) the sequence coverage of each pool (36.9x and 33.1x) was still relatively high and linkage was also strong (FET p-value < 1e-8).

Recombination rates also impact genetic resolution. *H13*, *H9*, and *H24* virulence are located near the telomeres of HF X chromosomes, where recombination frequency is extremely high. Mapping attempts resolved virulence to each of these *R* genes to a single candidate *Avr* gene. Attempts to map autosomal virulence, where recombination rates are much reduced, either disappointed (Behura et al., 2004), or failed (*H7H8*; Stuart, unpublished). Here, in comparison with the previous approach, BSA-seq efficiently improved the resolution of *H5* virulence on HF autosome A2 using the same mapping population used in a previous investigation (Behura et al., 2004). Recombination rates are typically low near the centromeres. Thus, we were impressed with how BSA-seq was able to resolve the position of *Hdic* virulence near the X2 centromere.

The power of BSA-seq to map genes with imperfect genomic sequenced maps was evident. Each experiment identified scaffolds that were partially linked to the genes in question, particularly among the “unmapped” scaffolds. Using the more conventional mapping approach, linkage between A1R.66 sequence and *Hdic* virulence would have required a much-improved HF genome assembly. Using BSA-seq, it was possible to detect this linkage in spite of the imperfect assembly.

Mapping *Hdic* virulence strengthened the hypothesis that the HF uses effectors to defeat basal plant immunity. Virulence to five *R* genes in wheat (*H6, H9, H13, H24,* and *Hdic*) has now been associated with one or more candidate *Avr* genes (**Table 1**). Each of these genes belongs to the “predicted effector” genic fraction of the HF genome (Zhao et al., 2015). Candidate *vH6* and candidate *vH9* are members of the largest family of putative effector-encoding genes (SSGP71) (Zhao et al., 2015). These genes appear to encode E3-ligase mimicking effectors. Candidate *vH24* is a member of another small family that appears to encode secreted phosphatase 2C effectors (Zhao et al., 2016). *vH13* is a unique gene that encodes a highly variable protein. Both candidate *vHdic* genes are members of the putative effector encoding SSGP4 gene family.

The present investigation also provides direct evidence that two *Avr*-encoded proteins have effector functionality in susceptible plant-parasite interactions: candidate-*vH6* and *vH13* suppressed the HR normally observed in *Nicotiana-B. glumae* interactions. Immune suppression is a well-established component of susceptible wheat-HF interaction. Infested susceptible wheat plants have lowered plant defense-related gene expression and reduced levels of defense-related phytohormones (Liu et al., 2007; Zhu et al., 2010). This inhibition is associated with the rapid development of the plant nutritive cells that are essential for HF larval survival (Harris et al., 2006; Subramanyam et al., 2018). The mechanisms underlying vH6 and vH13 immune suppression remain unknown.

Candidate-*vHdic* failed to suppress HR in the *Nicotiana*-*B. glumae* infiltration experiments. It is possible that the wrong candidate-*vHdic* was chosen for these experiments. It is also possible that this protein has lost its ability to suppress plant immunity. However, this observation does not eliminate the possibility that it is an effector protein. It simply may target other wheat physiological processes, or its target may not be intracellular. Moreover, Bacteria T3SS-based delivery, like any other heterologous method, has limitations. The machinery for protein synthesis in prokaryotes does not have the ability for post-translational modifications, which limits the analysis to eukaryotic effectors that do not require these modifications (Fabro et al., 2011).

The capacity of *B. glumae* to deliver eukaryotic T3SS-fusion effectors into plant cells expressed in pEDV system has been demonstrated previously and used to identify *M. oryzae* effectors with HR-suppressing effects in rice (Sharma et al., 2013). Suppression of *B. glumae*-induced HR in *N. benthamiana* has been used recently to identify several novel eukaryotic candidate effectors from the fungal pathogens *U. virens* and *L. theobromae* (Zhang et al., 2014), and the root knot nematode *M. incognita* (Shi et al., 2018a; Shi et al., 2018b). Here, we have added the HF to this list of eukaryotic plant pathogens and parasites. We anticipate that this system will be used to test hundreds of potential HF effectors for their effects on plant immunity.

## Data Availability Statement

Whole-genome sequencing data for bulked samples are available at the NCBI Sequence Read Archive (SRA) under the NCBI Bioproject accession number PRJNA613640 (https://www.ncbi.nlm.nih.gov/bioproject/?term=PRJNA613640).

## Author Contributions

LN-E performed BSA-seq and plant immunity experiments, designed experiments, analyzed data, and contributed to writing and editing the manuscript. CZ developed mapping populations, performed genetic mapping using microsatellite markers and analyzed data. RS analyzed data and made conceptual contributions. JS led the project, analyzed data and contributed to writing and editing the manuscript. All authors contributed to the article and approved the submitted version.

## Funding

The authors gratefully acknowledge support for this work provided by USDA-NIFA AFRI Grants 2008-35302-18816 and 2010-03741, Hatch award IND011462 to JS, USDA-ARS Specific Agreement 58-3602-4-010, and a fellowship to JS from Fulbright-Colombia.

## Conflict of Interest

The authors declare that the research was conducted in the absence of any commercial or financial relationships that could be construed as a potential conflict of interest.

The handling editor declared a shared affiliation with one of the authors CZ at the time of the review.
