# Supplementary material for: BSA-Seq Discovery and Functional Analysis of Candidate Hessian Fly (Mayetiola destructor) Avirulence Genes
Source: Front Plant Sci. 2020 Jun 25;11:956. doi: 10.3389/fpls.2020.00956 (PMC7330099; doi:10.3389/fpls.2020.00956)
Supplement: Supplementary file 1 [file DataSheet_1.docx]

**Supplementary Materials**

**Supplementary Tables**

**Table S1**. Whole genome high-throughput sequencing statistics

| **Bulk** | **N^a^** | **PE reads^b^** | **Total seq. (Gb)** | **Coverage** | **Mapped reads (%)^c^** | **Total SNPs** |
| --- | --- | --- | --- | --- | --- | --- |
| *H6*-avirulent | 19 | 27,530,110 | 2.72 | 14.6 x | 65.40 | 1.5 million |
| *H6*-virulent | 23 | 26,352,574 | 2.60 | 14.0 x | 65.77 |  |
| *Hdic*-avirulent | 15 | 31,180,664 | 3.07 | 16.5 x | 76.85 | 1.2 million |
| *Hdic*-virulent* | 33 | 92,713,232 | 9.14 | 49.2 x | 78.39 |  |
| *H5*-avirulent* | 24 | 69,360,376 | 6.85 | 36.9 x | 61.16 | 0.92 million |
| *H5-*virulent* | 24 | 62,368,904 | 6.16 | 33.1 x | 62.82 |  |

^a^ Number of individuals composing each bulk.

^b^Total number of paired-end (PE) reads after quality-filtering.

^c^ Percentage of reads mapped to reference genome after filtering for read-mapping quality.

* Represents merged PE libraries from 2 bulk-replicates.

**Table S2**. Primers sequences of the PCR-based markers used in this study.

| **Marker** | **Primer forward** | **Primer reverse** |
| --- | --- | --- |
| A1R66-169 | GAAAGCAACCAACATCGTGA | TGCGTAGCTTTATTTGGTGG |
| A1R66-4 | TCAACCAATAAGCCGACAAC | GATTACTGCGCCGGTGTAT |
| A1R66-62KB | TTTTCTTTTGGATATCGGTG | ATGGAAGTTTTGTCAAATGC |
| A1R66-85 | CTATCATCCGATTCATTCCG | GTCATGCTCTTGACGCATTC |
| A2.4-324 | CCATTAGAATTGTCATCAGC | ATAATATGCACGGTATGTCG |
| A2.5-402 | GTCTACTCATGCAAGAAATG | GTGTACACGAAATACACGA |
| A2.6-186 | GTATGACGTATGGCTGAA | CCTGATTCTTTGTGTCCAT |
| A2.6-189 | AGAACTGTCAAAGTCGATG | AACTCTTTGATGAGCTCTG |
| A2.7-201 | ATAGTGAACAATGGACACAC | TTAAACTCACTCTGAGAACC |
| A2.7-206 | CGAAATTGCTGTACTGAAC | TATTATCTACACCGATTCCC |
| A2.9-262 | ATTTCGTATGTACACCACAC | GTGACTACGATTGTGATGAT |
| X2.7-2 | TGTGTTCTGTGTCTAGCTGC | ACCGTTTTGGATGAAGTATG |
| X2.7-21 | TTCTCACTTTCATCGGCTAT | AGACGAAACAAGGAACAAAA |
| X2.8-163 | AACATCAACATCAACAAGCA | ATTCGTCAGCTTATTACCGA |
| X2.8-190 | GACGAAAGCAATTGAACTTT | CGAATATCGAATATCGAAGG |
| X2.8-195 | CATCCAGCCTCTTTCTAATG | GTCTGGGTATTGAAGTTGGA |
| X2.8-2.3MB | CCTTTGTGTCGCAAAATTTC | TTTCTTTGTTCGGTTTTTCG |
| X2.8-202 | ACGATTCAGGAATTTATCCTC | GGCAAATACATTATGAACAG |
| X2.8-215 | TTAGCTTTGTGATAAACGCA | TATAATGATGTATGGCAGCG |
| X2.8-221 | GATACCTTAATCGGCATGAA | GCGAGACATAAACAATGGAT |

**Table S3**. RT-PCR gene-specific primers

| Gene^a^ | Forward primer | Reverse primer |
| --- | --- | --- |
| Mdes007166-RA | ATGAAAGTGTTCATTTGCGTTGTTT | TTAAACTAGAACGGTGGCACCAAAAG |
| Mdes007165-RA | ATGAAAGTGTTGATTTGCGTTGTTC | TCAAATCAAAATGGTTTTGGTTAC |
| Mdes007227-RA | ATGGCAAAAGATGGAATTTTTCTAA | TTATTTAAGTTCAATCGAATCCGTG |
| Mdes011440-RA | ATGAATTTACAATTTTTATTGTTAG | CTATACATCATCTTTTGAGACCTC |
| Mdes007205-RA* | GCTTGGCTGTTGCCCTTATC | CCCCATCTTACGGCCTGTTT |
| Mdes007142-RA(a)* | ATGTCTATCAAAGTTACTTTGGGTT | TTAATGTCCTGGTTTTTTATCTAAG |
| Mdes007142-RA(b) | CCATCAGCAGTTGGTTCAGC | GGCACTTGGAATGGCTGTTG |
| Mdes007107-RA | GTAAGTGCTGCCCAGGCATA | AGCACCAGGTACGTCATTCG |
| Mdes007160-RA | TGTTGCATTGTGCGTTCCAG | TTTGTTGCTAACACAGCCCC |
| Mdes007161-RA | TTGACCGTTGCTGTTCCAGT | ACCAGTCGATGGATAGGTGC |
| Mdes007167-RA | TTAGCTGCAATTGCTACCGC | TTTTTGAAACGGACACCGCC |
| Mdes004090-RA | ACAGCTTGGCTGTGATCCTG | CCGTAAGCTCTGGTGCAACT |
| Mdes004116-RA | GATTTGCCGACGTGTTGCAT | GACGCATATTTGGGCGTTCC |
| Mdes005935-RA | TGCACTCTAACCAACTTGCCA | CCAGTGGGTTTTCCATTGCC |
| Mdes005938-RA | CCGTTCCTTTTGTTGTGGGC | CGGGTCCTCTGAAATCCGTT |
| Mdes005941-RA | GGGACCGTACAGCAATGACA | GCCACCGCTATCTCCACTAC |
| Mdes005949-RA* | ATCGGCAGTGTCCAACCATT | TGCACGCACAGTGAACAAAA |
| Mdes005952-RA* | CGACGAGGTAAAGGTGCCAA | CAAGAAACGCTTTCACGCCT |
| Mdes005963-RA | GCCAAGTTTACGATGGGCAC | CTGCTGCTTTCCATGGTGAC |
| Mdes005968-RA* | AGCCGCACCATTAGATCCAG | CCTTTGCGGCCTTCGATTTG |
| Mdes004160-RA* | CGATATCAGGCTTTATGAAGG | TTTGGCATGATTAATAGATC |
| Actin | AGCCAACAGAGAAAAGATG | ACCAGCCAAATCCAAAC |

^a^Names in the official gene set (OGS), which can be accessed at the USDA Arthropod i5k official workspace <https://i5k.nal.usda.gov/data/Arthropoda/maydes-(Mayetiola_destructor)/GCA_000149185.1/> and the genome assembly curated at the National Center for Biotechnology Information (NCBI), GenBank assembly accession number GCA_000149185.1

<https://www.ncbi.nlm.nih.gov/assembly/GCA_000149185.1>

Synonymous names for these gene models follow: Mdes007205-RA = SSGP71-137, Mdes007142-RA(a) = SSGP47-1, Mdes005949-RA = SSGP4-12, Mdes005952-RA = SSGP4-13, Mdes005968-RA = SSGP4-14 and Mdes004160-RA = SSGP4-60.

**Table S4**. Primer list for Gateway cloning

| Primer | Sequence^a^ | Vector^b^ |
| --- | --- | --- |
| attB1vH6For | GGGGACAAGTTTGTACAAAAAAGCAGGCTTCGACGGTGGGGCTGCAACTTCATCA | pDONR221 |
| attB2vH6Rev | GGGGACCACTTTGTACAAGAAAGCTGGGTCTTATTTCTTACAAGACTTGCA | pDONR221 |
| attB1vH6CFor | GGGGACAAGTTTGTACAAAAAAGCAGGCTTCTTCCTGATATTCGGCAAC | pDONR221 |
| VH13 GW F | CACCTCTCCACTTCCTCTGGC | pENTR/TEV/D-TOPO |
| VH13 GW R | TTATTTGGCTTTCTTTTTTTTAG | pENTR/TEV/D-TOPO |
| vHdic cd2 GW-F | CACCGAACGAATTATCACTGAA | pENTR/TEV/D-TOPO |
| vHdic cd2 GW-R | TTACGGGATGCACGGAATAC | pENTR/TEV/D-TOPO |

^a^ Underlined nucleotides correspond to the Gateway adaptors. ^b^ Gateway entry vector used for gene cloning.

**Table S5**. Microsatellite markers used for mapping *Hdic*-virulence

| Marker | Scaffold | Position^a^ | Recombiants^b^ |
| --- | --- | --- | --- |
| X2.7-2 | X2.7 | 49433 | 12 |
| X2.7-21 | X2.7 | 380695 | 12 |
| X2.8-163 | X2.8 | 1506605 | 3 |
| X2.8-190 | X2.8 | 1937330 | 2 |
| X2.8-195 | X2.8 | 2037097 | 2 |
| X2.8-202 | X2.8 | 2138414 | 2 |
| X2.8-215 | X2.8 | 2247940 | 2 |
| X2.8-221 | X2.8 | 2325397 | 0 |
| X2.8-2.3MB | X2.8 | 2351355 | 0 |
| A1R66-4 | A1Random.66 | 12809 | 0 |
| A1R66-62KB | A1Random.66 | 62998 | 0 |
| A1R66-85 | A1Random.66 | 589005 | 0 |
| A1R66-169 | A1Random.66 | 1040500 | 4 |

^a^ Scaffold position (base) based on Hessian fly reference genome version Mdes 1.0.

^b^ Number of recombinant individuals out of 48 total in the vHdic-RILF10 mapping population.

**Table S6**. Genes on regions of A1R.66 and X2.8 linked to *Hdic*-virulence

| **Gene^a^** | **Position** | **Protein length (aa)** | **Best BLASTP hit^b^** | **Locus^c^** | **E-value** |
| --- | --- | --- | --- | --- | --- |
| Mdes005935-RA | A1R.66: 188037 | 107 | Hypothetical protein [Safg] | WP037672913.1 | 1.3 |
| Mdes005938-RA | A1R.66: 207653 | 394 | CLIP-domain serine protease subfamily C [Adar] | ETN65690.1 | 3.0E-87 |
| Mdes005941-RA | A1R.66: 218589 | 353 | Trypsin-8 [Nlug] | AID60342.1 | 5.0E-113 |
| Mdes005949-RA | A1R.66: 259363 | 192 | Protein Shroom3 [Oafe] | XP007947231.1 | 1.3 |
| Mdes005952-RA | A1R.66: 298946 | 221 | Tail tube protein (P1-like gp21) [Vvul] | WP 032071943.1 | 1.1 |
| Mdes005963-RA | A1R.66: 422183 | 453 | Hypothetical protein FF3812479 [Lcup] | KNC32340.1 | 2.0E-149 |
| Mdes005968-RA | A1R.66: 631495 | 209 | O-succinylbenzoate–CoA ligase [Vmet] | AAT09151.1 | 6.2 |
| Mdes004160-RA | X2.8: 2349877 | 193 | Respiratory burst oxidase-like protein 3 [Rcor] | AKS03956.1 | 2.2 |

^a^Names in the official gene set (OGS), which can be accessed at the USDA Arthropod i5k official workspace <https://i5k.nal.usda.gov/data/Arthropoda/maydes-(Mayetiola_destructor)/GCA_000149185.1/> and the genome assembly curated at the National Center for Biotechnology Information (NCBI), GenBank assembly accession number GCA_000149185.1

<https://www.ncbi.nlm.nih.gov/assembly/GCA_000149185.1>

^b^ Streptomyces afghaniensis [Safg], Anopheles darling [Adar], Nilaparvata lugens [Nlug], Orycteropus afer afer [Oafe], Vibrio metoecus [Vmet], Vibrio vulnificus [Vvul], Lucilia cuprina [Lcup], Rubia cordifolia [Rcor].

^c^ Sequence ID for the best BLASTP hit in NCBI databases.

**Table S7**. Microsatellite markers used for mapping *H5*-virulence

| Marker | Scaffold | Position^a^ | Recombinants^~~b~~^ |
| --- | --- | --- | --- |
| A2.4-147 | A2.4 | 1667960 | 5 |
| A2.4-324 | A2.4 | 3334451 | 7 |
| A2.5-341 | A2.5 | 375292 | 6 |
| A2.5-402 | A2.5 | 1318322 | 1 |
| A2.6-186 | A2.6 | 58611 | 0 |
| A2.6-189 | A2.6 | 111881 | 0 |
| A2.7-201 | A2.7 | 593360 | 0 |
| A2.7-206 | A2.7 | 830330 | 0 |
| A2.9-262 | A2.9 | 1757537 | 1 |

^a^ Scaffold position (base) based on Hessian fly reference genome version Mdes 1.0.

^b^ Number of recombinant individuals out of 36 total in the *vH5*-RILF2 mapping-population.

**Supplementary figures:**


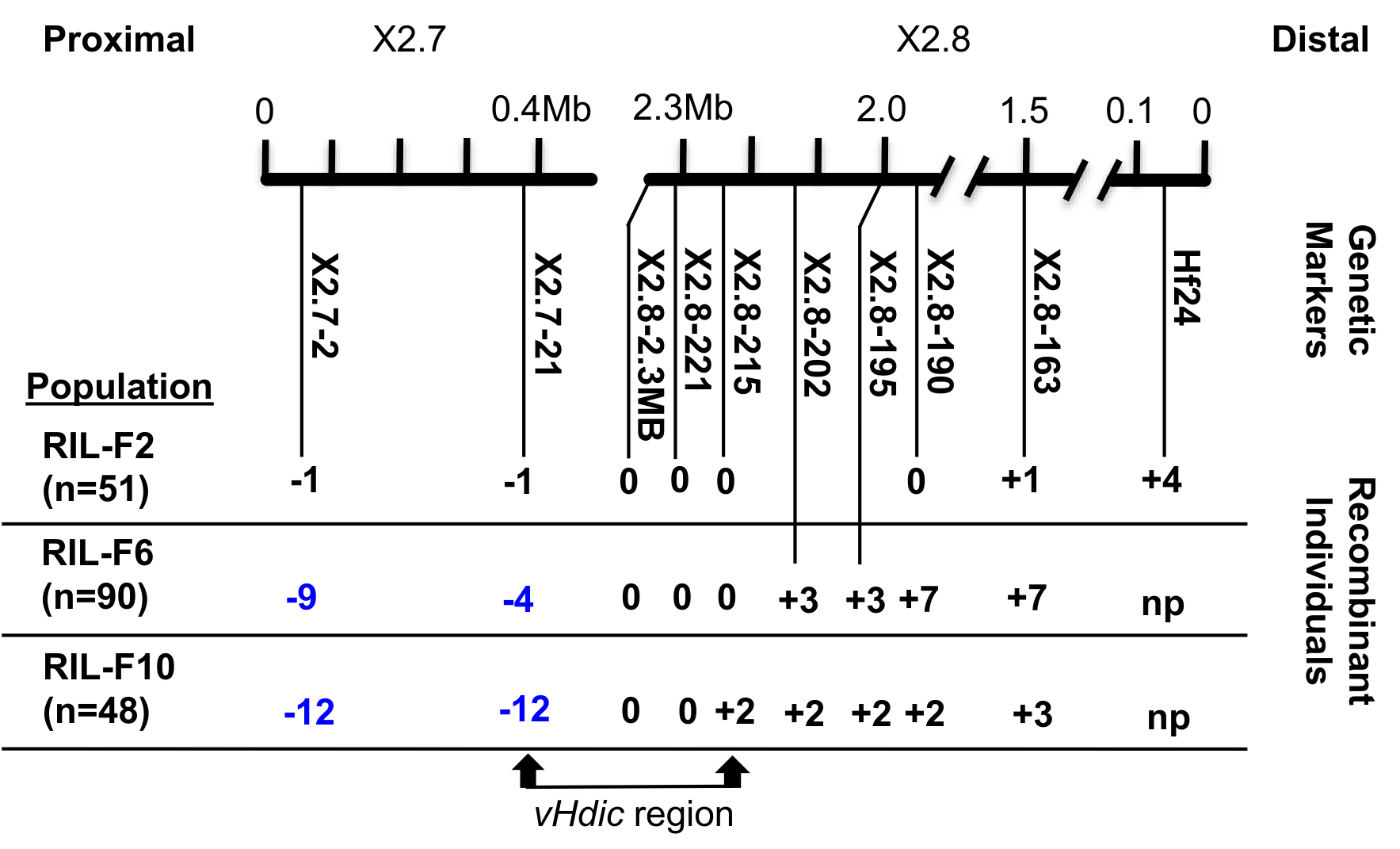


**Figure S1**. *vHdic* mapping using PCR-based markers. *Hdic*-avirulent and *Hdic*-virulent males from generations F2, F6 and F10 in the *vHdic*-RIL population were genotyped with PCR-based genetic markers on scaffolds X2.7 and X2.8. Number of recombinant individuals are shown for each generation. Blue numbers correspond to recombinants from only avirulent individuals.

>Mdes004160-RA SSGP4-60

MSNSRKLIIFCLLFAVIWVQSLKAERIITENDIRLYEGQMLLASTHNYIPNHFNSLGSWEYISDEVKQKIVQIYFEMLDKIVCRNDEEKYEAQIKSSYAALDSFIKNELEKIRNIFNEDPKVCQMIELEEKGKFLEYLAFTSYNQSEIQKDLKKKKKKSLIYTDDDLLIMANVNEYLHYVAKSGSRNCIPCIP

>Mdes005968-RA SSGP4-14

MLNSQKFIIFCLLFAVQSLKAAPLDPEQAKLIGQFERAITYSASDDESQFRIHLNSWGSWRKIPDQLKIKIALRYDAELKKLSMSQSHIPMKTKRYFAHKTLNTFIKNDLLQIVKELYALDPKSKAAKEMKKVQNNGESLDYFAFIHDQSQLPAIEAELEILKKDGIKYVKDQARIVAYVNAYLEWRDNAKANAKASGSRSIFGQCFSC

**Figure S2**. Protein sequences of best *vHdic* candidate genes. Underlined sequences indicate the secretion signal peptides as predicted by SignalP 4.1 server (<http://www.cbs.dtu.dk/services/SignalP-4.1/>).


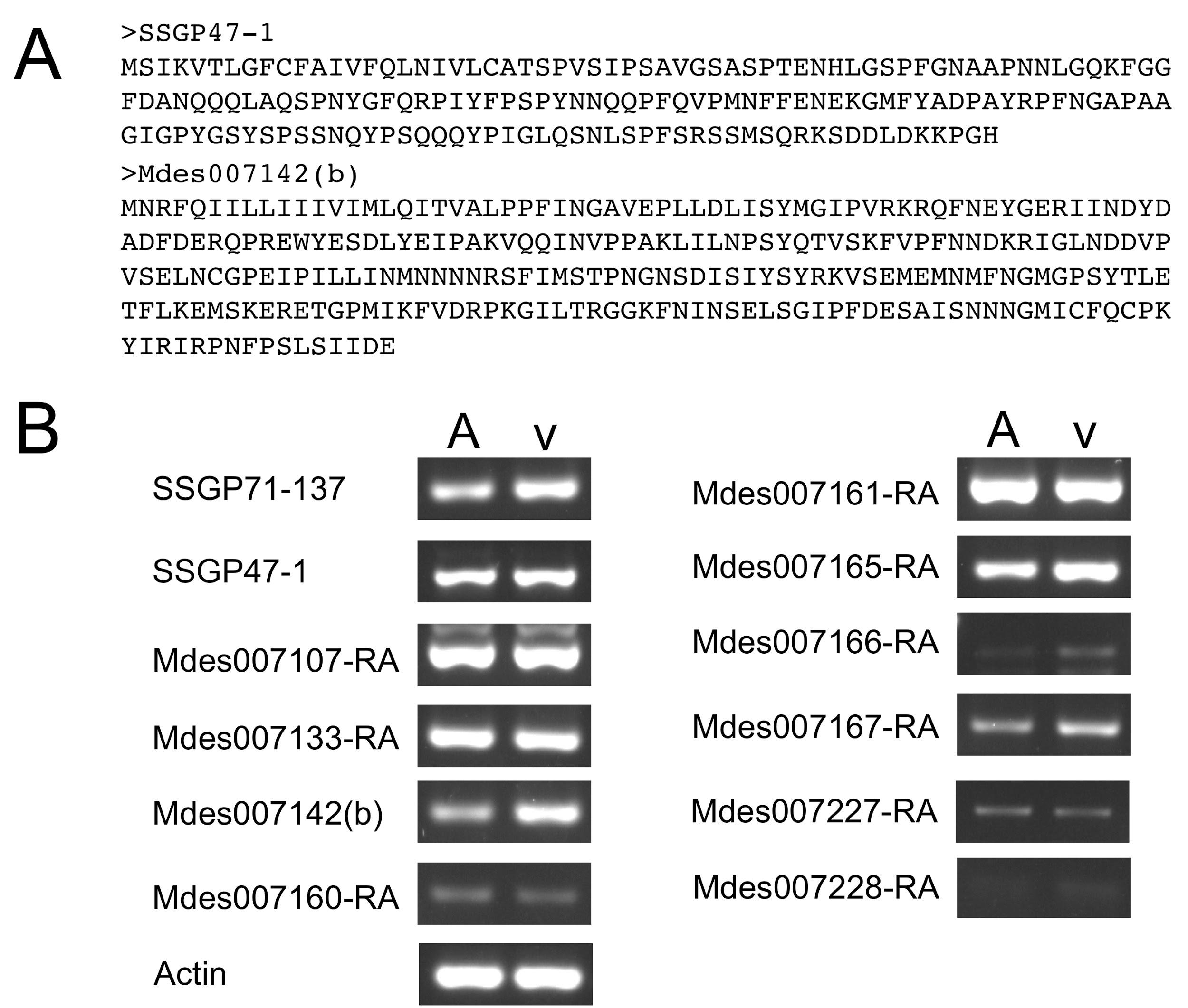


**Figure S3.** (A) Predicted proteins SSGP47-1 and Mdes007142(b) discovered within the original OGS gene model Mdes007142-RA. Mdes007142(b) was predicted using the on-line Ab initio gene prediction Fgenesh (<http://www.softberry.com/>). (B) Reverse transcription PCR (RT-PCR) for genes encoding signal-peptides containing proteins within the *H5*-virulence linked scaffold A2.7. Similar results were obtained in each of three independent biological replications. Putative effector gene SSGP71-137 corresponds to Mdes007205-RA. Mdes gene IDs corresponde to names in the official gene set (OGS), which can be accessed at the USDA Arthropod i5k official workspace <https://i5k.nal.usda.gov/data/Arthropoda/maydes-(Mayetiola_destructor)/GCA_000149185.1/>.
